# The Molecular Cytogenetic Characterization of Pistachio (*Pistacia vera* L.) Suggests the Arrest of Recombination in the Largest Heteropycnotic Pair HC1

**DOI:** 10.1371/journal.pone.0143861

**Published:** 2015-12-03

**Authors:** Pedro J. Sola-Campoy, Francisca Robles, Trude Schwarzacher, Carmelo Ruiz Rejón, Roberto de la Herrán, Rafael Navajas-Pérez

**Affiliations:** 1 Departamento de Genética, Universidad de Granada, Campus de Fuentenueva s/n, 18071, Granada, Spain; 2 Department of Biology, University of Leicester, University Road, Leicester, LE1 7RH, United Kingdom; CNRS/University Lyon 1, FRANCE

## Abstract

This paper represents the first molecular cytogenetic characterization of the strictly dioecious pistachio tree (*Pistacia vera* L.). The karyotype was characterized by fluorescent *in situ* hybridization (FISH) with probes for 5S and 45S rDNAs, and the pistachio specific satellite DNAs PIVE-40, and PIVE-180, together with DAPI-staining. PIVE-180 has a monomeric unit of 176–178 bp and high sequence homology between family members; PIVE-40 has a 43 bp consensus monomeric unit, and is most likely arranged in higher order repeats (HORs) of two units. The *P*. *vera* genome is highly heterochromatic, and prominent DAPI positive blocks are detected in most chromosomes. Despite the difficulty in classifying chromosomes according to morphology, 10 out of 15 pairs (*2n = 30*) could be distinguished by their unique banding patterns using a combination of FISH probes. Significantly, the largest pair, designated HC1, is strongly heteropycnotic, shows differential condensation, and has massive enrichment in PIVE-40 repeats. There are two types of HC1 chromosomes (type-I and type-II) with differing PIVE-40 hybridization signal. Only type-I/II heterozygotes and type-I homozygotes individuals were found. We speculate that the differentiation between the two HC1 chromosomes is due to suppression of homologous recombination at meiosis, reinforced by the presence of PIVE-40 HORs and differences in PIVE-40 abundance. This would be compatible with a ZW sex-determination system in the pistachio tree.

## Introduction

Nuts of pistachio (*Pistacia vera* L., Anacardiaceae) have recently reached the fifth position worldwide in nut production, with around 920k tons harvested in 2013 [1]. Male and female varieties are cultivated and asexually reproduced, with ‘Peter’ (a male cultivar) and ‘Kerman’ (a female cultivar) being among the most widespread [2]. Pistachio trees, as all other members of the genus, are strictly dioecious. The floral buds, although potentially bisexual, suffer selective abortion of flower organs during flower development, giving rise to male and female plants [3].

Although dioecy is common in bryophytes and gymnosperms, this condition is rare in angiosperms, occurring only in 5–6%of them [4–5]. Contrary to the situation in animals, where gender separation is normally mediated by a sex-chromosome system, only a few tens of plant species have distinguishable sex chromosomes. There is cytogenetic and/or molecular evidence for the presence of sex chromosomes in 0.01% of angiosperms, 0.6% of gymnosperms, and 0.02% of bryophytes [6]. Convergent evolution of sex chromosomes implies the accumulation of several sex-determining genes and sex-antagonistic genes (sex-specific beneficial alleles that can be neutral or even disadvantageous for the other sex) in a pair of standard autosomes, followed by the arrest of recombination which leads to Y-chromosome molecular degeneration [7–9].

Satellite DNAs tend to accumulate in regions where recombination rates are low [10]. Their role in the erosion of non-recombining Y chromosomes has been well documented in several groups of plants, namely *Rumex* [11], *Silene* [12], and papaya [13]. The involvement of rDNAs in sex-chromosome differentiation has also been demonstrated. Both the *Marchantia polymorpha* L. (common liverwort) and *Spinacia oleracea* L. (spinach) X chromosomes carry a cluster of 45S, whereas no rDNA is found on their Y-chromosome counterparts [14–15]. The location of rDNA and repetitive sequences, combined with DAPI staining, provide useful markers for chromosome identification, and has been previously used for the karyotype characterization of other plants with sex chromosomes, such as *Rumex* sp. [11], *Coccinia grandis* [16], *Humulus japonicus* [17], and *Cannabis sativa* [18].

The genus *Pistacia* contains at least 11 species [19]. The few cytological studies performed to date reveal that the chromosome number of *P*. *vera* L. and *P*. *terebinthus* L. is *2n = 30* [20–21], as is the case of *P*. *khinjuk* Stocks [22], *P*. *lentiscus* L. [23], *P*. *integerrima* L. [24], and *P*. *eurycarpa* Yalt. [21]. However, Ghaffari and Fasihi-Harandi [25] later proposed a chromosome number of *2n = 24* for *P*. *lentiscus* and *P*. *khinjuk*. The *2n = 28* chromosome number of *P*. *atlantica* Desf., reported by Zohary [26], would be *2n = 30*, according to Ila et al. [21]. The only report for *P*. *chinensis* Bunge described a chromosome number of *2n = 28* [27]. All these data would imply three different basic chromosome numbers for the group, *n = 12*, *n = 14* and *n = 15* [28]. Such discrepancies in chromosome counting have been imputed to the reduced size of *Pistacia* chromosomes and to the fact that only a few cell divisions are visible per root tip. Al-Saghir [29] considering molecular, morphological, and cytogenetic data proposed that all *Pistacia* species would have a basic chromosome number of *n = 15*.

Recently, several sex-linked SNP markers have been found to be heterogametic in pistachio females suggesting a ZZ/ZW sex determination [30]. In fact, there is general agreement that the largest pair of chromosomes is heteropycnotic at least in the members of the group for which cytological analyses have been performed, including *P*. *vera*, *P*. *atlantica*, and *P*. *khinjuk* [20, 28]. Not without controversy, the involvement of this pair in sex determination has been proposed [31]. Given the dioecious condition of all *Pistacia* species, the 1:1 sex ratio of pistachio trees, and considering that the pair meets the two requirements of most plant-sex-chromosomes described so far (to be the longest or nearly longest pair of the chromosome complement and to be highly heterochromatic -[6]), suggests a sex-chromosome-based system with genetic determination through a single locus in *Pistacia* [32]. However, not many cytological or molecular studies have been carried out to date in *Pistacia*, and the molecular composition and organization of this chromosome pair remain unknown.

In this context, the present study constitutes the first molecular cytogenetic characterization of pistachio. For this, we have carried out DAPI banding and FISH using probes of rDNA and two satellite-DNA families, isolated by NGS technologies, and described here for the first time. We propose *P*. *vera* to become a promising model to study dioecism evolution.

## Materials and Methods

### 1. Plant material and DNA isolation

*Pistacia vera* L. male (‘Peter’) and female (‘Kerman’) leaves for DNA isolation, and nuts for mitotic analysis were harvested from the research center ‘El Chaparrillo’ (Ciudad Real, Spain). Leaves were quickly frozen at -20°C for genomic DNA isolation using the Invisorb® Spin Plant Mini Kit (STRATEC Molecular GmbH, Berlin, Germany). The quality of genomic DNA was measured by Infinite® 200 PRO NanoQuant (Tecan, Switzerland) and confirmed by electrophoresis on a 1% agarose gel.

### 2. Identification, isolation, and characterization of satellite-DNA families

Two approaches were followed to detect new satellite DNAs in *P*. *vera*: (1) by digesting total genomic DNA using different restriction endonucleases, and (2) by exploring 454 libraries.

For restriction analyses, 10 μg of genomic DNA were digested with a battery of restriction enzymes following the manufacturer’s recommendations (Roche). After electrophoresis, the restriction products which showed a ladder pattern were excised from agarose gels and purified with IlustraTM GTXTM PCR DNA and Gel Band Purification Kit (GE Healthcare). After purification, bands were ligated to pGEM®-Teasy cloning vector (Promega). After the selection of transformant colonies, inserts were extracted by PCR and sequenced in an Applied Biosystems sequencer as described elsewhere [33].

Two NGS 454 runs were carried out, from male (105,277 reads) and female (82,143 reads), consisting of 95,023 total contigs spanning 41,001,719 bp (~3.6% of the estimated whole genome length taken as reference the size of closely related *P*. *terebinthus* -1115Mbp [34]). The outputs containing the sequence and the quality data were merged into a.fastq file and trimmed by quality (Q30). For exploration, the raw reads from male and female were trimmed in 200 bp, and used as input for RepeatExplorer Galaxy-based software to establish sequence clustering [35]. Clusters were selected according to sequence abundance (>3% of the total 454 sequences).

Then, the contigs of each cluster were extracted, aligned and processed with the program Geneious 6.1.7 (http://www.geneious.com/-Biomatters). For further analysis, specific primers for satellite-DNA amplification (PIVE-40 F: CTCTAGTCACTATCGGGTG PIVE-40 R: TCATGAGGAACGGGTAGG, PIVE-180 F:AGCCAACATAGTCGATCTCGT PIVE-180 R: GCTTTCTGCTCGTTTTGTCA) were designed using Primer3 (http://bioinfo.ut.ee/primer3-0.4.0/ -[36]. Variability between and within sequences of different cultivars and phylogenetic analyses were conducted using MEGA 5.2 [37]. Trees were constructed by the neighbor-joining method [38]. Thousand bootstrap replicates [39] were performed to assess internal support for nodes. Rates of change were analyzed with satDNA Analyzer software [40].

### 3. DNA quantification

The relative quantity of both satellite-DNA repeats described here was measured by iCycler Thermal Cycler with iQ5 Multicolor Real-Time PCR Detection System (BIORAD). PCR reactions were performed in 25 μl containing 100 ng of template DNA, 1X of the iQ™ SYBR® Green Supermix (BIORAD), 10 pmol of each primer, and ddH_2_O up to final volume. The reactions were carried out under the following conditions: 95°C for 3 min, followed by 35 cycles at 95°C for 30 sec, 64°C for 30 sec, 72°C for 45 sec, and final extension at 72°C for 7 min. The resulting data were obtained through iQ5 software (BIORAD). An analysis of variance factor (Stat / ANOVA / One-Way), followed by an analysis of mean square deviation (MSD) post hoc for multiple comparisons were applied in both satellite DNA to test differences between males and females in quantitative PCR results. Differences were tested to P = 0.01 and P = 0.05. Additionally, the percentages of each repetitive sequences found in 454 reads were used to estimate the relative genome abundance of satellite DNAs in male and female.

### 4. Chromosome preparations, DNA probes, and Fluorescent *in situ* hybridization (FISH)

Twenty-three seeds from an open-pollinated ‘Kerman’ cultivar were germinated in autoclaved fine sand soaked in distilled water until roots emerged. Root tips of unsexed specimens were excised and treated in 0.002M 8-hydroxyquinoline for 4h at RT and fixed in 3:1 (v/v) absolute ethanol: glacial acetic acid. To obtain metaphasic chromosomes, root-tip spreads were prepared using pectinase (15 U/ml) and cellulase (80 U/ml) digestion prior to squashing in 45% acetic acid onto cleaned microscope slides as described by Schwarzacher et al. [41]. In each experiment, at least 20 mitotic metaphase plates were analyzed.

The following probes were used: PIVE-40 repeat and PIVE-180 repeat, both obtained by PCR on genomic DNA using specific primers, ITS region of 45 rDNA (amplified using primers in [42]) and 5S rDNA (amplified using primers in [43]). PCR reactions were performed as described elsewhere [33]. All probes used were labeled with both digoxigenin-11-dUTP and biotin-11-dUTP using random priming kit, BioPrime® Array CGH Genomic Labeling System (Invitrogen), following manufacturer’s instructions. FISH experiments were performed as described by Schwarzacher and Heslop-Harrison [44]. The chromosomes were counterstained with 4 μg/ml of DAPI (4’, 6’-diamidino-2-phenylindole) and mounted in antifade solution. A Zeiss Axioplan 2 epifluorescence microscope (Oberkochen, Germany) was used to observe chromosome preparations. The metaphase plates with fluorescent signals were photographed using suitable filters with a CCD camera (Optronics, model s97790). The chromosome number was determined in several cells from each individual. Karyogrammes were made attending to size, morphology, banding, and position of centromere. Colour figures and overlays were prepared with Adobe Photoshop 7.0 software, using only those processing functions that are applied to all pixels of the image.

## Results

### 1. Isolation of PIVE-180 satellite-DNA family in *P*. *vera* by restriction analysis

**Fig 1:**
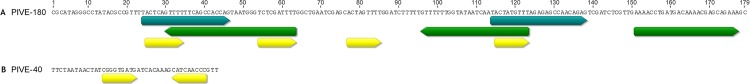
Consensus sequence of monomeric units. Consensus of PIVE-180 (A) and PIVE-40 (B), showing direct repeats (green), inverted repeats (blue), and short repeats (yellow).

When total genomic DNA of *P*. *vera* was digested with HinfI restriction enzyme and electrophoresed in agarose gels stained with ethidium bromide, several prominent bands of 200-bp fold units were observed. Thus, 200- and 400-bp fragments were excised and cloned as candidate repeat units of a satellite-DNA family. The 400-bp band was hybridized against genomic DNA of both male and female varieties of *P*. *vera* ('Peter' and 'Kerman', respectively) digested with different restriction enzymes and blotted onto a nylon filter. As a result, lanes digested with HinfI and TaqI showed a typical ladder pattern with a repeat unit of 200 bp. This would support the contention of a tandem organization for these repeats (**S1 Fig**). Twenty candidate clones were then sequenced. Sequences showed homology between them but no significant positive matches were detected when any of those sequences was BLASTed against Genbank (originally in March, 2014 and again in January, 2015), and then considered a new satellite-DNA family (see consensus sequence, in **Fig 1A**), from now on PIVE-180 (**PI**stacia **VE**ra **180**-bp repeat). No other tandemly arrayed repeat was found following this method.

### 2. Isolation of PIVE-40 satellite-DNA family in *P*. *vera* by exploring 454 libraries

When the two 454 libraries were exploited for repeats two clusters of satellite sequences were found to represent more than 3% of total reads. One of them corresponded to the PIVE-180 family, described above. The other one showed no homology, either with PIVE-180 or when BLASTed against Genbank and was named PIVE-40 (see consensus sequence, in **Fig 1B**).

### 3. Presence and relative abundance of satellite-DNA families

Distribution and organization of both PIVE repeats were investigated in male and female individuals by PCR using specific primers. Both repeats gave a positive A-ladder amplification, confirming their presence in both varieties and the tandem organization, characteristic of satellite-DNA sequences. Relative abundance of PIVE sequences between males and females was assessed by quantitative PCR. Only PIVE-40 showed significant differences to P = 0.05 between sexes, being more represented in males.

Sequence number comparisons in the 454 reads of males and females showed significant differences for PIVE-180 (3.79% and 3.05% of number of 454 reads for males and females, respectively) and PIVE-40 (3.93% and 3.38% for males and females, respectively) between male and female libraries (P<0.001). NGS sequence quantification often underestimates the amount of repeats. It could explain the discrepancies observed between this method and qPCR for PIVE-180.

### 4. Molecular characterization and variability analyses of satellite-DNA families

In the case of PIVE-180, a set of 131 complete monomeric units were analyzed. Of these, 20 were isolated by PCR (EMBL accession numbers LN831332-LN831351), cloning and sequencing from males (12) and females (8) individuals, while 111 belonged to the 454 reads (EMBL accession numbers LN868813-LN868923) of males (39) and females (72). Their size ranged from 176 to 178 bp, and the AT average content was 59%. Two repeated motifs of 23 bp with 69.6% of identity, and three direct repeats (one of these, complementary sequence) about 18 bp, with a mean identity of 67%, were visible in the consensus sequence (**Fig 1A**). A variability study using the entire dataset showed a degree of identity among monomeric sequences of 83.9%. A NJ tree did not reveal the existence of highly supported groups of sequences according to their sex (**S2 Fig**). A comparative study of male and female sequences revealed that 53.11% of the positions were identical in the two sexes and no fixed diagnostic positions were found between them.

In the case of PIVE-40, a set of 232 sequences (EMBL accession numbers LN868680-LN868812), (126 and 106 for males and females, respectively) were extracted from the 454 reads. The consensus monomeric unit had 43 bp in length, and an AT average content of 60.5%. A motif of 9 bp was found directly and inversely repeated (**Fig 1B**). An intraspecific variability study using the whole dataset (male and female sequences) revealed a total identity value of 74.2%. A NJ tree showed different clusters of sequences. Further analysis revealed that in most cases (about 60%), consecutive units from the same read, did not group together (**S3 Fig**), but instead showed the existence of two different types of consecutive monomers. The variability within each type was around 89% in both cases, while the divergence between them was 68.5%. No sex-related clustering was found (**S3 Fig**).

### 5. Description of *P*. *vera* karyotype using DAPI staining, and PIVE-40, PIVE-180, 5S and 45S rDNA probes

**Fig 2:**
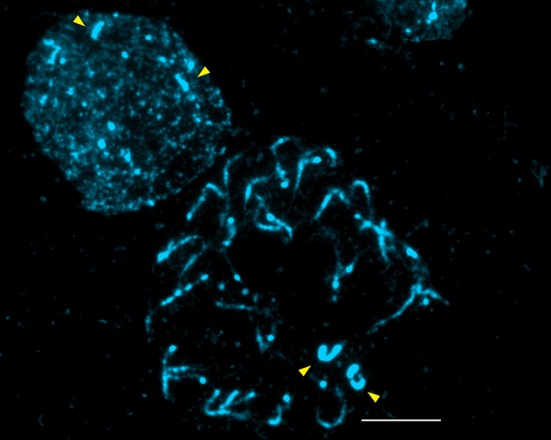
Cells showing differential condensation of HC1 pair. Root tip interphase (top left) and pro-metaphase (middle) of *P*. *vera*, (*2n = 30*) after DAPI staining. The differential condensation of HC1 pair is evidenced by large DAPI positive signal at interphase and bright, relatively short and compact chromosomes at pro-metaphase (arrow heads) while the smaller DAPI positive chromocentres at interphase are from the centromeres of the other chromosomes that are considerably longer at pro-metaphase. Bar represents 2.5 μm.

**Fig 3:**
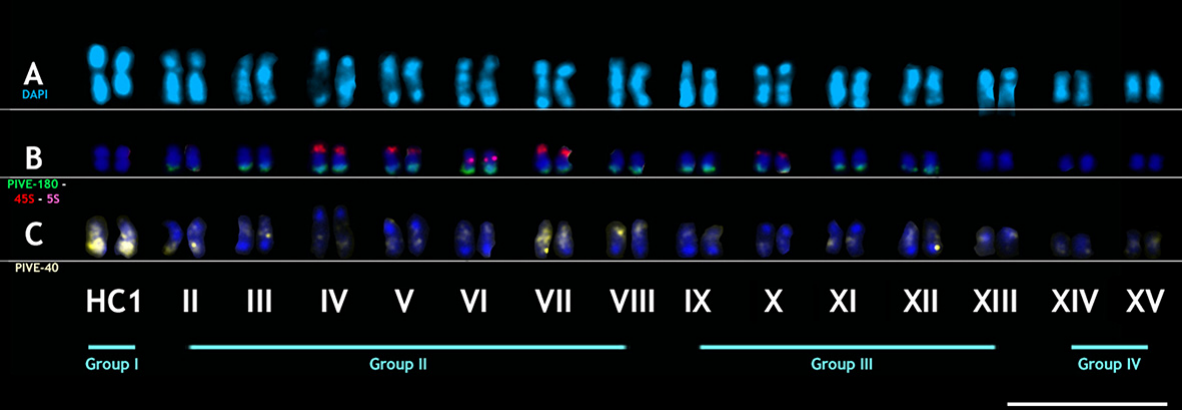
Karyotype of *P*. *vera*. Karyotype root-tip metaphase chromosomes of *P*. *vera* (n = 15) stained with DAPI (A) showing two larger chromosomes (designated HC1) that are strongly heterochromatic. *In situ* hybridization using ribosomal DNA probes (red, 45S rDNA, pink, 5S rDNA), PIVE-180 (green) (B), and PIVE-40 (yellow, C). Bar represents 2.5 μm.

The mitotic-chromosome complement in growing apical cells of this species has *2n = 30* chromosomes with chromosomes ranging from 0.45 μm to 0.88 μm. Multiple DAPI positive bands were visible at intercalary positions, and at the ends of most chromosomes, suggesting that the pistachio genome is largely heterochromatic. In prometaphase, a pair of chromosomes appears differentially condensed (**Fig 2**, arrows) and was later designated as the largest heteropycnotic HC1 pair (see **Fig 3**). Except for this clearly distinguishable pair, the rest of chromosomes are difficult to classify using morphology only. Therefore, four different FISH probes were used to assist the karyotyping.

**Fig 4:**
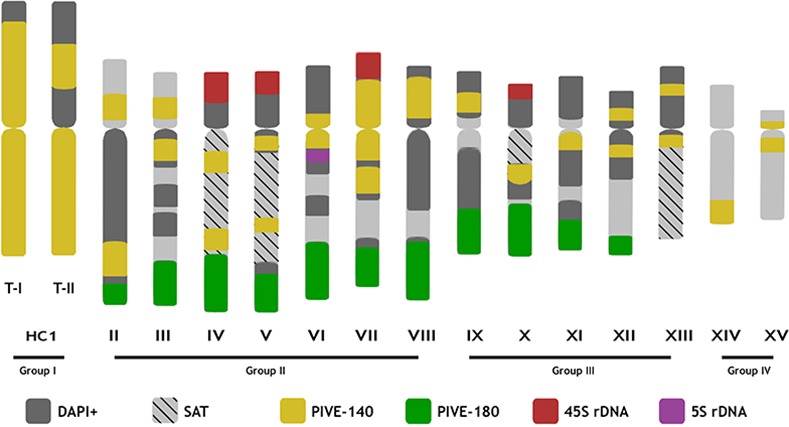
Idiogram of haploid chromosome complement of *P*. *vera*. PIVE-40, PIVE-180, and 5S and 45S rDNA sites are shown. DAPI + regions and satellite chromosomes are also indicated. Chromosome types and sizes were constructed from the estimation of the centromere position.

FISH using rDNA probes showed that 45S rDNA sequences are present in eight loci in terminal positions of the short arms of chromosome pairs IV, V, VII, and X (**Fig 3B**, red, and **Fig 4**and **S4 Fig**), while 5S rDNA consisted of two loci in pair VI at intercalary positions (**Fig 3B**, pink, and **Fig 4**, and **S4 Fig**).

PIVE-180 probes showed prominent clusters in terminal regions of the long arm in 11 pairs (II to XII), but not in pairs XIII to XV. Significantly, the HC1 pair also lacked PIVE-180 hybridization signal (**Figs 3B** and **4**, green, and **S4 Fig**). On the contrary, PIVE-40 was highly accumulated in the HC1 pair, being also ubiquitous in all chromosomes at different positions: in pairs III, VI, VII, XI-XIII, and XV, it exhibited a pericentromeric location; in pairs IV, V, and X, it was located at intercalary positions of the long arm; in pairs VIII and IX, it was at intercalary positions of the short arm; in pair II, it was located at intercalary positions of both long and short arms; and in pair XIV, it was located at the end of the long arm (**Figs 3C** and **4**and **S4 Fig**).

According to these results the karyotype can be arranged in four groups: the first group is constituted exclusively by the conspicuous largest pair of DAPI+/heteropycnotic metacentric chromosomes (HC1 –heteropycnotic chromosome I). The second group is represented by seven similarly sized pairs of submetacentric chromosomes (pairs II to VIII). The third group includes five pairs of submetacentric chromosomes of similar size (IX to XIII), while the fourth group is formed by a pair of submetacentric (XIV) and acrocentric (XV) small chromosomes (**Figs 3**and **4**).

Two chromosomes were found to carry 5S rDNA loci and with similar size and morphology can be considered homologous chromosomes. Eight chromosomes, paired into four pairs of homologous, carried the 45S rDNA loci. Three of these were associated with a pair of SAT chromosomes (satellite chromosomes) (see chromosome pair IV, V and X, **Fig 3A**). PIVE-180 repeats were present at subterminal positions of the long arms of chromosome pairs II to XII, but were absent in the remaining three pairs (**Fig 3B**). PIVE-40 repeats are accumulated massively in the HC1 pair, and are also present at subterminal, pericentromeric, and intercalary positions in the rest of *P*. *vera* chromosomes (**Fig 3C**).

**Fig 5:**
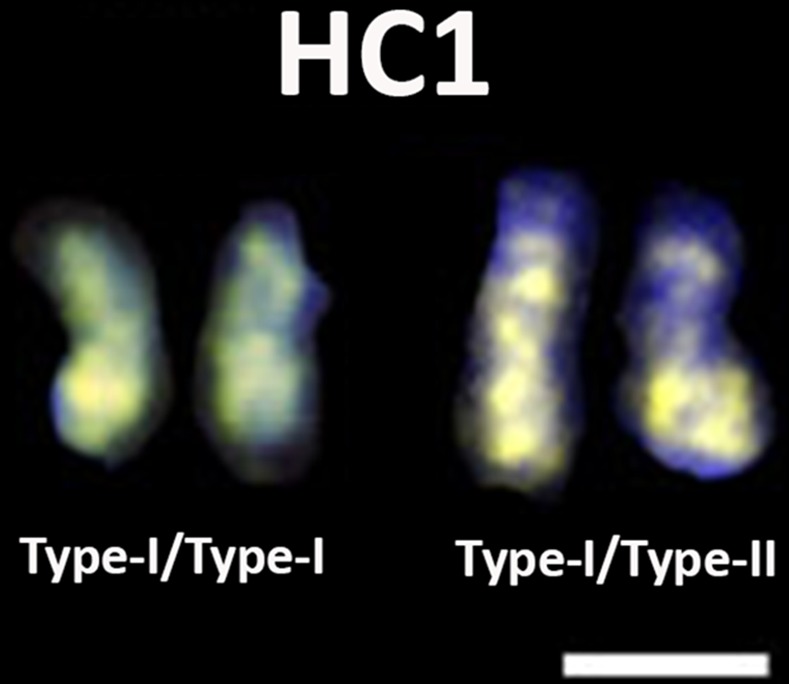
Two types of HC1 chromosomes attending to PIVE-40 distribution. Differential distribution of PIVE-40 repeats in the mitotic HC1 pair of *P*. *vera*. Bar represents 1 μm.

Significantly, two different patterns of PIVE-40 were found in the HC1 pair (**Figs 4**and **5**). Type-I chromosomes were characterized by a strong signal along most of both arms while type-II chromosomes exhibited one of the arms with two faint signals, the other one remaining as in type-I pattern. Heterozygotes and type-I homozygotes were equally detected among the individuals analyzed in this paper. No type-II homozygotes were found.

## Discussion

### 1. Molecular cytogenetic characterization of pistachio

The cytogenetic characterization of members of the genus *Pistacia* poses a challenge due to the difficulty of classifying the chromosomes according to their morphology. Our results using 5S and 45S rDNAs, as well as PIVE-40 and PIVE-180 satellite DNAs as probes together with DAPI counterstaining demonstrate that the karyotype of pistachios is *2n = 30*, and can be arranged into four groups of chromosomes according to chromosome size, morphology, position of centromeres, banding and DAPI staining (**Fig 3**). Ten of the 15 pairs of pistachio chromosomes could be specifically identified from the other chromosomes and from each other (**Fig 4**).

The estimation of sequence abundance supports a higher accumulation of PIVE-40 and PIVE-180 in males, although in all cases the abundance felt below 4%. Satellite DNAs can represent up to 20% of plant nuclear DNA [45]. However, the indirect procedures of measurement, such as the one used in this paper, can often underestimate the quantity or repetitive DNA [46].

### 2. Chromosome and rDNA loci evolution in *Pistacia*

We report here for the first time the number and location of rDNA loci for *P*. *vera*. Our data update the results in Harandi and Ghaffari [47] who using conventional techniques of chromosome observation, hypothesized that up to five chromosome pairs are associated with the nucleolus in diakinesis. Apart from that report, only two records of rDNA distribution in other Anacardiaceae species are found in the literature. De Souza Almeida et al. [48] determined the presence of two loci of 5S, and two loci of 45S in different *Spondias* species (*2n = 32*). Yonemori et al. [49] observed two loci of 5S, and six loci of 45S in mango (*Mangifera indica* L.– *2n = 40*). Thus, the number of 5S loci has remained constant in Anarcardiaceae, being interstitial in *P*. *vera*, and terminal in the other species. The location of the 45S loci is terminal in all three species, however, the number of loci varies, with two loci in *Spondias* sp., six loci in mango, and eight loci in pistachio. There is no evidence of recent whole genome duplications from the data available up to this date, but due to the low number of studies it cannot be ruled out.

### 3. Evolutionary dynamics of PIVE repeats

PIVE families show several repetitive motifs within the monomers. Non-adjacent inverted repeats and palindromic sequences have been associated with DNA amplification and recombination processes [50]. Additionally, monomeric units could have arisen from amplification of minor repetitive units as has been reported in animals, e.g. the satellite DNAs of *Mus musculus* [51], *Sparus aurata* [52], *Oreochromis mossambicus*, *O*. *hornorum* [53], *Dicologlossa cuneata* [54] and *Drosophila buzzatii* group [55], but also in plants, *Rumex acetosa* [33].

A preferential satellite-DNA monomer length between 150–180 bp has been found in many centromeric satellite DNAs. It corresponds in length to the 1.67 turns around the histone octamer core (147 bp) plus the linker DNA between the nucleosomes, typically varying 10–70 bp (reviewed in [56]). PIVE-180 has a repetitive unit of 180 bp, close to the estimated mean size in plants of 165 pb [57]. However, PIVE-180 is located in the distal region of the chromosomes and has not been found near the centromeres as many 180-bp satellite DNAs in other plant species have. In plants, the subterminal region has been proved more variable and polymorphic than any other region of the genome, and is often occupied by satellite DNAs [58–59]. The accumulation of repeats in this region is thought to facilitate meiotic pairing, protect terminal genes loss or substitute telomere function when this disappears [60]. Due to the proximity with the telomeres sometimes degenerated telomeric repeats are found intermixed [61]. This is not the case of PIVE-180, for which no telomeric motifs were found. The variability analysis of PIVE-180 sequences showed high levels of identity between sequences (83.9%), within males (85.3%) and females (83.7%), similar to other satellite DNAs [33]. This indicates that the processes of recombination and homogenization mechanisms are operating normally and that the chromosome interchange is not impeded for PIVE-180.

PIVE-40 repeats, against the principle of heterochromatin equilocality, are widespread throughout the *P*. *vera* genome. Additionally, they show an unusual monomeric size (43 bp). Satellite DNAs with similar repetitive unit size have been reported in several species such as the 35-pb satellite DNA of *Scilla siberic*a [62], the 24-bp family of *Musca domestica* [63] or the 44-bp one of *Ceratitis capitata* [64]. The approach taken in this study, using long 454 sequences to characterize repetitive sequences, has allowed us to analyze contiguous repeated units. The NJ analysis showed that non-contiguous repeats of PIVE-40 tend to cluster. In fact, about 60% of PIVE-40 sequences showed higher degree of identity with non-contiguous repeats than with contiguous ones (**S3 Fig**). This suggests a dynamic of dimeric higher-order repeats (HORs) for these sequences, which tend towards longer arrays, a typical evolutionary mechanism of satellite-DNA amplification [65]. These HORs are found in both male and female genomes. Its existence has been associated with regions of low recombination rates [45]. This is true of the beetle *Pholeuon proserpinae* satellite DNA [66] or the centromeric alpha satellites in mammals [67]. Also, clusters of HORs of the human alpha satellite differ and show autosomal and sex-chromosome specificity [67–68]. The presence of HORs in PIVE-40 could be due to the pericentromeric location of a portion of these sequences. However, it is not negligible that the vast majority of PIVE-40 sequences are accumulated in the HC1 pair (as demonstrated by FISH, **Figs 3**and **4**), proposed as a possible sexual pair. Extreme cases of lack of recombination are represented by specific satellite DNA in sex chromosomes [69].

### 4. Sex determination in pistachio

Sex determination in pistachio could be mediated by the presence of a pair of sex chromosomes, as proposed by several authors, based on the presence of a heteropycnotic pair, the largest in the complement of several *Pistacia* species [28, 31, 47], and sex-linked SNP markers [30]. That candidate pair corresponds to the pair I of heterochromatic chromosomes (HC1) described in this paper (**Figs 3**, **4**and **5**).

Sex chromosomes are thought to have evolved from a standard autosomal chromosome pair as a consequence of a rarely recombining region that contains sex-determining and antagonistic genes involved in sex determination, followed by arrest of recombination, and Y-chromosome degeneration [7]. Apart from the amplification of transposable elements (mainly LTR retrotransposons), that is considered one of the major forces contributing to the large size of plant Y chromosomes [70–71], it is well known that the accumulation of tandemly-arrayed repetitive sequences (microsatellites and satellite DNAs) has triggered the differentiation of sex chromosomes in several plant species [6, 9, 69, 72]. In fact, Y chromosomes of most dioecious plants exhibit chromatin expansion, are the largest (or nearly) chromosomes in the complement, and show a morphological differentiation with respect to X counterparts. This is true of heteromorphic sex chromosomes of *Rumex suffruticosus* [11] or *Silene latifolia* [12]. In other cases, heteromorphy can be caused by the smaller size of the Y chromosomes, as in *Humulus lupulus*, which might have undergone deletion of parts of the MSY region (male-specific region of the Y chromosome) [18] or *Humulus japonicus*, probably because of X-autosome translocations [17].

In an attempt to clarify the structure of HC1 pair of pistachio, 5S and 45S rDNAs, and PIVE-40 and PIVE-180 satellite DNAs were physically located by FISH. As mentioned above, no evidence of presence of rDNA was detected in this chromosome pair.

As for the satellite-DNA families, PIVE-40 and PIVE-180 were more represented in the genome of males than in females of pistachio. Their chromosomal distribution proved different as well (**Fig 3**). Remarkably, while PIVE-180 was completely absent from the HC1 pair, PIVE-40 was massively accumulated on this pair. Considering that probes penetrated equally in all chromosomes, the presence of two types of HC1 chromosomes with respect to the distribution of PIVE-40 signals has been showed here (**Fig 5**). Differentiation between sex chromosomes of *Cannabis sativa*, *H*. *lupulus*, and *H*. *japonicus* has been demonstrated with regard to the differential location of the subtelomeric repeats CS-1, HSR-1, and HJSR of, respectively [18]. Also, the differential accumulation of RAYSI and RAE180 satellite-DNA repeats demonstrates chromosomal rearrangements between Y_1_ and Y_2_ sex chromosomes of several *Rumex* species [73].

PIVE-40 accumulation in the HC1 pair, and the possible presence of HORs in this location might be contributing to the molecular differentiation between the two members of the HC1 pair as a consequence of a non-recombining region. This is supported by the existence of type-I and type-II chromosomes(**Fig 5**). Type-I homozygotes and type-I/type-II heterozygotes have been detected, but not type-II homozygotes. This data together with the fact that PIVE-40 is more represented in males would support a ZZ (type-I homozygotes)/ZW (type-I/type-II heterozygotes) system as previously described in pistachio by Kafkas et al. [30]. Unfortunately, we were unable to determine the gender of the individuals carrying heteromorphic HC1 pairs because the first blooming lags around 5–7 years, and seeds of unknown sex were used in our analyses so HC1 chromosome types cannot be unambiguously related to sex determination. Sex-specific markers have only been available very recently in pistachio [30]. These markers are promising, and will contribute to improve studies as the one performed here, but still unable to separate sexes in all cases, as in wild species (*P*. *atlantica* (Desf.), *P*. *terebinthus* L., *P*. *eurycarpa* Yalt., and *P*. *integerrima* Stewart).

Despite the differential accumulation of PIVE-40, no strong evidence of morphological differentiation of HC1 pair in mitosis was found using a molecular cytogenetic approach (**Fig 3**). This is supported by the meiotic chromosome dynamic in pollen mother cells of male pistachio trees consisting of 15 similarly sized bivalents [47]. A mean number of chiasmata of 1.35 for each bivalent at the first meiotic metaphase has been reported, with mainly terminal chiasmata, and only infrequent interstitial chiasmata [28, 47]. This would promote the absence of homogenization. Although, there is a relation between the molecular erosion of Y/Z chromosomes and heteromorphy, we found some exceptions in the literature. The best studied model is papaya (*Carica papaya* L.). Despite the fact that its MSY region has expanded some 134% with respect to its X counterpart [74], sex chromosomes show no evidence of morphological differentiation [75–76].

Taking into account that HC1 is strongly heteropycnotic, differentially condensed in prometaphase, enriched in at least a family of satellite DNA, and with some level of heteromorphy at molecular level, we could hypothesize its involvement in sex determination in pistachio. However, our study needs to be completed with further analyses. Kafkas et al. [30] proposed the existence of a ZW sex-determining system still in an early stage of differentiation similar to that described for papaya based on the presence of heterozygous SNPs markers in pistachio females. It is therefore of interest to test whether SNPs markers are located in the *P*. *vera* HC1 pair, and if it is homologous with the heteropycnotic pair of other *Pistacia* species, and above all, to finally confirm the involvement of this pair in the sex determination of this group of species. The completion of the draft genome sequence and the genetic map based on microsatellites of this species could enable this in the near future. Our research is currently focusing on these aspects.

## Supporting Information

S1 Fig Southern-blot hybridization using PIVE-180 probes. Kerman (lanes 1, 3, 5, 7, and 9) and Peter (2, 4, 6, and 8) total genomic DNA cut with EcoRI (1, 2), RsaI (3, 4), HinfI (5, 6), TaqI (7, 8), and AluI (9, 10) using the monomeric PIVE-180 satellite DNA sequences as probe. (*), (**), (***) indicate monomer, dimer, and trimer, respectively. (M), (F) mean male and female, respectively.
(TIF)

S2 Fig Neighbor-joining tree based on PIVE-180 sequences. Numbers at each node indicate bootstrap support. Sequences from female genomic DNA start with FEM; and from male DNA with MAL.
(TIF)

S3 Fig Neighbor-joining tree based on PIVE-40 sequences. Numbers at each node indicate bootstrap support. Sequences from female genomic DNA start with FEM; and from male DNA with MAL. In red sequences that showed higher homology with non-contiguous sequences than with contiguous ones (in blue).
(TIF)

S4 Fig FISH in root-tip metaphase chromosomes of *P*. *vera* (n = 15) counterstained with DAPI. 45S rDNA (A), 5S rDNA, red, and PIVE-180, green (B), and PIVE-40 (C). Bar represents 2.5 μm.
(TIF)
