# Supplementary figures and images for: The Molecular Cytogenetic Characterization of Pistachio (Pistacia vera L.) Suggests the Arrest of Recombination in the Largest Heteropycnotic Pair HC1
Source: PLoS One. 2015 Dec 3;10(12):e0143861. doi: 10.1371/journal.pone.0143861 (PMC4669136; doi:10.1371/journal.pone.0143861)

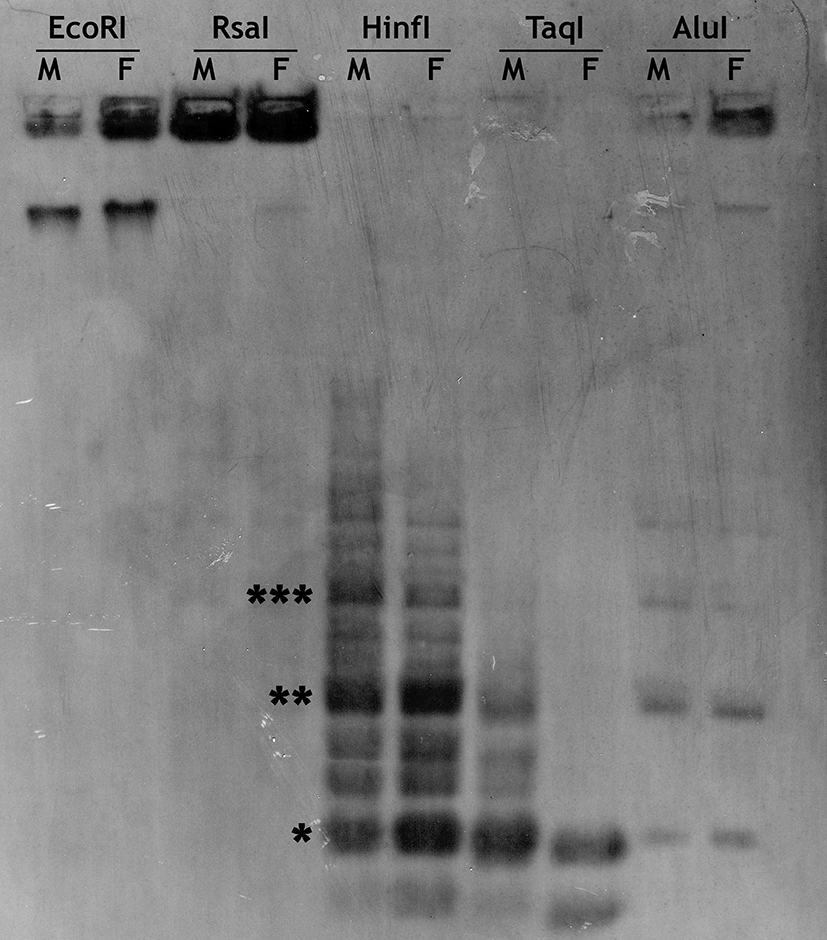

Supplement: S1 Fig — Kerman (lanes 1, 3, 5, 7, and 9) and Peter (2, 4, 6, and 8) total genomic DNA cut with EcoRI (1, 2), RsaI (3, 4), HinfI (5, 6), TaqI (7, 8), and AluI (9, 10) using the monomeric PIVE-180 satellite DNA sequences as probe. (*), (**), (***) indicate monomer, dimer, and trimer, respectively. (M), (F) mean male and female, respectively. (TIF) [file pone.0143861.s001.tif]

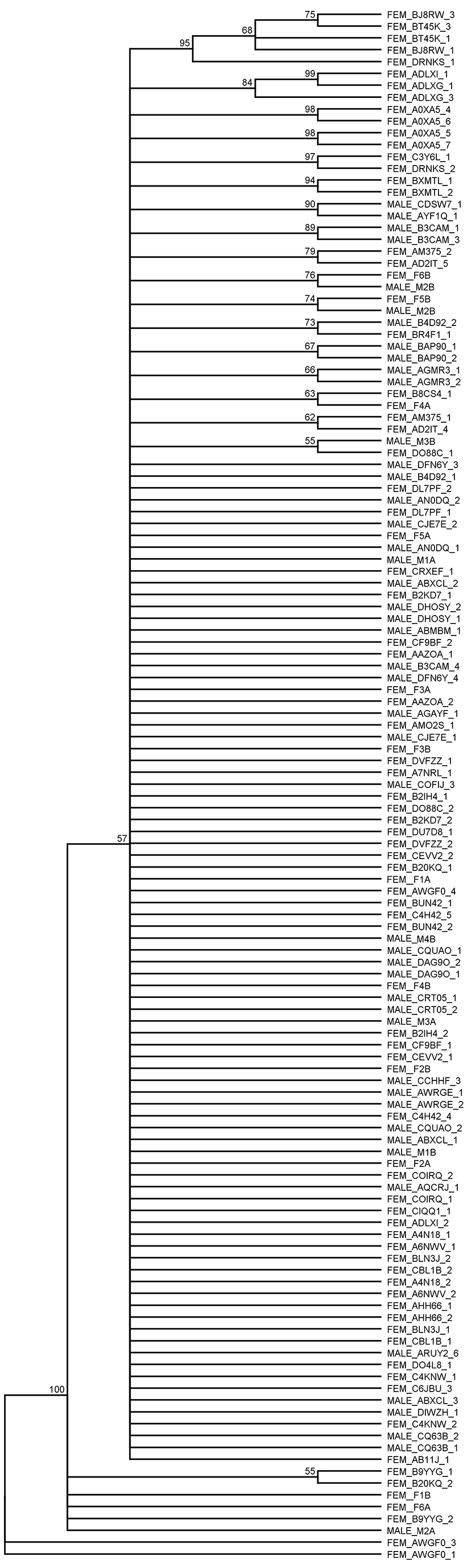

Supplement: S2 Fig — Numbers at each node indicate bootstrap support. Sequences from female genomic DNA start with FEM; and from male DNA with MAL. (TIF) [file pone.0143861.s002.tif]

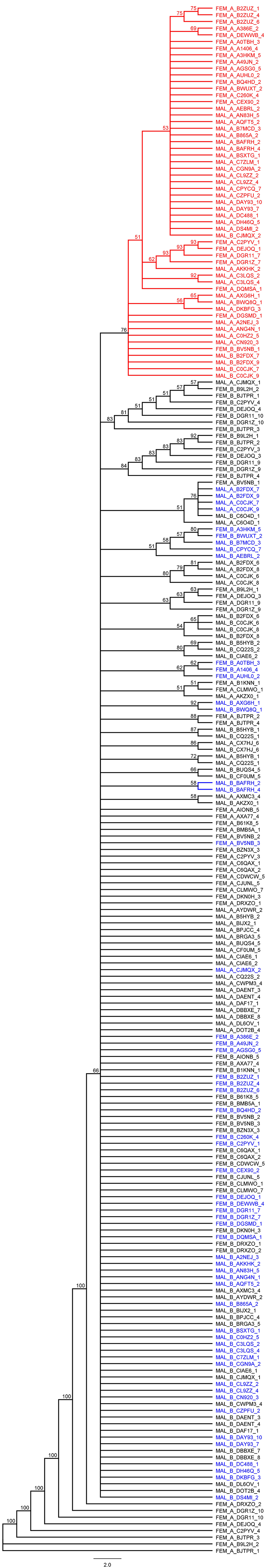

Supplement: S3 Fig — Numbers at each node indicate bootstrap support. Sequences from female genomic DNA start with FEM; and from male DNA with MAL. In red sequences that showed higher homology with non-contiguous sequences than with contiguous ones (in blue). (TIF) [file pone.0143861.s003.tif]

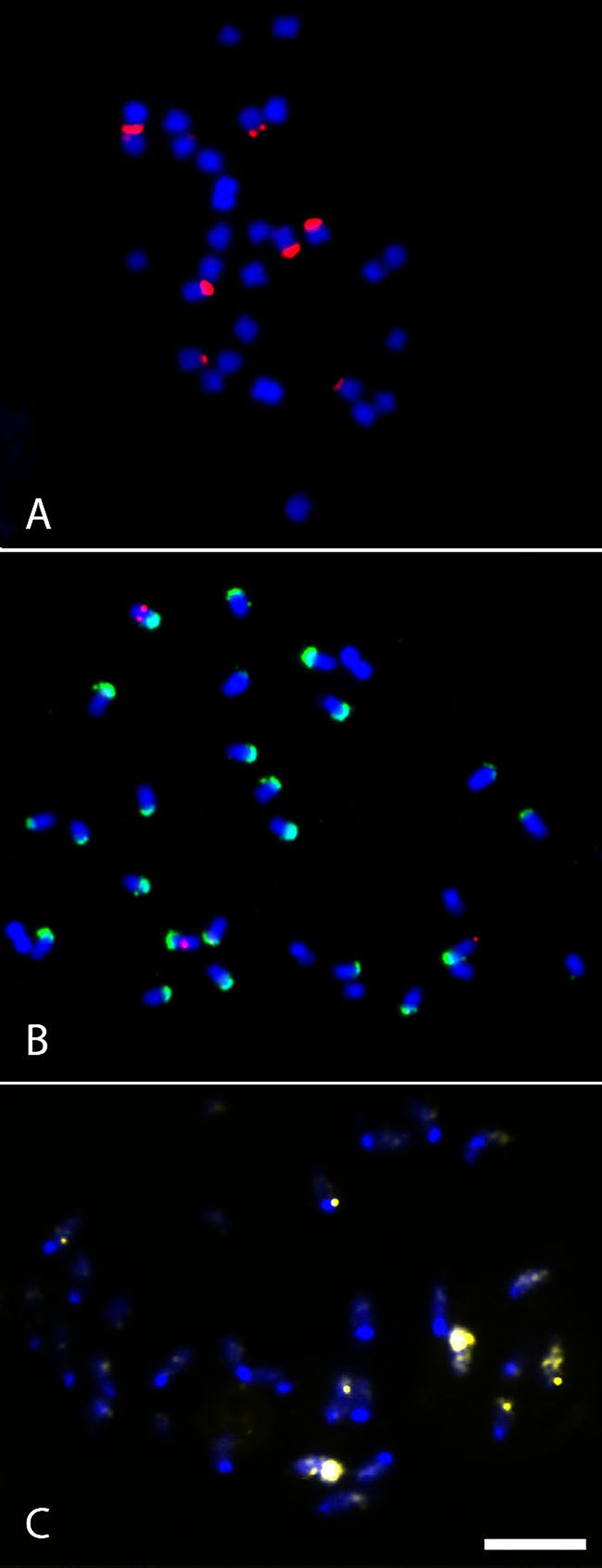

Supplement: S4 Fig — 45S rDNA (A), 5S rDNA, red, and PIVE-180, green (B), and PIVE-40 (C). Bar represents 2.5 μm. (TIF) [file pone.0143861.s004.tif]
